# When mesophotic meets shallow: First records of deep‐associated reef fishes in a well‐studied tropical bay under the influence of spring–summer upwelling

**DOI:** 10.1111/jfb.70592

**Published:** 2026-07-22

**Authors:** Miguel Loiola, Rodrigo Maia‐Nogueira, Carla Sthefany Barroso Teles, Maisha Gragnolati, Guilherme Camargo Lessa, Pedro Milet Meirelles, Igor Cristino Silva Cruz, José Amorim Reis‐Filho

**Affiliations:** ^1^ Institute of Biology Federal University of Bahia Salvador Brazil; ^2^ Long‐Term Ecological Research Program of the Baía de Todos os Santos (PELD TSB) Federal University of Bahia Salvador Brazil; ^3^ Biological Oceanography Laboratory (LOB) Federal University of Bahia Salvador Brazil; ^4^ Department of Biological Science State University of Bahia (UNEB) Alagoinhas Brazil; ^5^ Reef System Ecology and Conservation Lab (LECAR) Fluminense Federal University Niterói Brazil; ^6^ Programa de Pós‐Graduação em Ecologia (PPGE) Universidade Federal do Rio de Janeiro Rio de Janeiro Brazil; ^7^ Departament of Oceanography Institute of Geosciences, Federal University of Bahia Salvador Brazil; ^8^ Instituto Nacional de Estudos Interdisciplinares e Transdisciplinares em Ecologia e Evolução (IN‐TREE) Salvador Bahia Brazil; ^9^ Marine Ecology and Conservation (MARES) Laboratory Instituto de Ciências do Mar (LABOMAR), Universidade Federal do Ceará (UFC) Fortaleza Brazil

**Keywords:** episodic cooling events, habitat permeability, rare ecological events, shelf–bay interactions, tropical reef dynamics

## Abstract

We document the first records of reef fishes typically associated with mesophotic environments on shallow (<10 m) coral reefs inside a well‐studied tropical bay. The vermilion snapper *Rhomboplites aurorubens* and the pygmy angelfish *Centropyge aurantonotus*, usually restricted to deeper (>40 m) shelf habitats along the Brazilian coast, were recorded inside Baía de Todos os Santos (BTS). Integrating long‐term ecological and oceanographic monitoring, fisheries information and underwater visual records, we show that these occurrences coincided with coastal upwelling and the advection of cooler shelf waters. We propose that cold‐water intrusions act as thermal corridors enabling cross‐shelf movements.

Cross‐shelf movements of fishes between mesophotic and shallow habitats, in both directions, are explained, at least in part, by a combination of environmental drivers and species‐specific life‐history strategies (Heidmann et al., 2024). Key structuring factors include depth, substrate type and structural complexity, hydrodynamic regime and cross‐shore distance, all of which shape habitat suitability across the continental shelf (Brokovich et al., 2006; McGehee, 1994; Silva et al., 2021). Investigations of cross‐shelf ontogenetic movements for several carnivorous, invertivorous, planktivorous and even herbivorous fishes (Fukunaga et al., 2016; Moura et al., 2011) have indicated that water depth is a dominant organizing variable that constrains strictly shallow‐affiliated species to nearshore habitats while maintaining mesophotic‐associated species at greater depths (Garcia‐Sais, 2010). This pattern, while seemingly intuitive, reflects a fundamental ecological principle: Species distributions along environmental gradients are not continuous but structured by ecological boundaries, physiological thresholds and habitat‐species performance trade‐offs (McKenzie et al., 2016; Reis‐Filho et al., 2019). Such boundaries emerge from the interaction between abiotic gradients and species‐specific biological requirements, generating sharp transitions in community composition and limiting the permeability of habitat interfaces (Chatfield et al., 2010). In this sense, the water depth operates not merely as a physical dimension but as an ecological filter that reinforces species segregation and high rates of species turnover across shelf gradients (Jankowski et al., 2015; Rocha et al., 2018).

Thermal stratification, both vertical and horizontal, represents an additional and often underappreciated mechanism reinforcing habitat segregation in coastal seascapes (Silva et al., 2025), particularly in tropical systems where temperature gradients interact with bathymetry to constrain species distributions (Samoilys et al., 2019). Deep‐associated fishes are therefore expected to remain within relatively narrow thermal envelopes and depth ranges that optimize physiological performance and ecological fitness (Baldwin & Robertson, 2014; Brokovich et al., 2008). Although cross‐shelf movements from deeper to shallower habitats are not inherently precluded (Ransier et al., 2024), the combined action of bathymetric gradients and temperature‐mediated limits typically maintains distinct fish assemblages between mesophotic and euphotic zones, even in regions characterized by high connectivity (but see Vergés et al., 2019). Against this background, we report unusual occurrences of two deep‐associated fish species, the vermilion snapper *Rhomboplites aurorubens* (Cuvier, 1829) (Lutjanidae) and the pygmy angelfish *Centropyge aurantonotus* Burgess, 1974 (Pomacanthidae), within the interior of Baía de Todos os Santos (BTS), a coastal bay with 9.8 m average depth and mean annual temperature of 27.7 ± 1.4°C (Lessa et al., 2019; Reis‐Filho et al., 2019). BTS has been designated as a multiple‐use protected area since 1999, although the absence of an implemented management plan has resulted in weak enforcement and limited governance effectiveness (Reis‐Filho et al., 2024). While such records would already be noteworthy in the context of updating the bay's ichthyofaunal inventory, one of the most diverse in the South Atlantic Ocean encompassing more than 400 identified fish species (Oliveira‐Silva et al., 2024), their ecological significance is substantially amplified by evidence from a long‐term ecological monitoring programme. This programme allowed us not only to document the presence of these species, but also to identify the timing of their entry into the bay and to link these events to periods of a seasonal thermal corridor associated with upwelling‐driven cooling of the adjacent shelf (Thévenin et al., 2019).

The species involved, the vermilion snapper and the pygmy angelfish, had never been recorded inside the bay throughout more than two decades of diverse fish studies, including intensive underwater surveys, systematic fishery monitoring, targeted research collections and curated scientific collections. These sources are summarized in Table 1 to provide historical context rather than as exploratory datasets for the present analysis, while the new records reported here are based on BRUVs and underwater visual census data. FishBase lists the vermilion snapper as occurring from 40 to 300 m depth, usually between 40 and 100 m, and the pygmy angelfish from 12 to 200 m depth (Froese & Pauly, 2026). Along the tropical eastern and northeastern Brazilian coast, the vermilion snapper is characteristically associated with mesophotic environments, typically occurring at depths exceeding 40 m and contributing primarily to small‐scale fisheries operating at deeper fishing grounds, often below 50 m (Freitas et al., 2011; Messias et al., 2019; Pereira et al., 2021). In contrast, at higher latitudes along the Brazilian coast, this species may occasionally occur in shallower habitats, a pattern likely facilitated by reduced thermal stratification and weaker separation between shallow and mesophotic zones (Anderson et al., 2025; Gragnolati et al., 2024). *C. aurantonotus*, in turn, is a well‐documented component of tropical southwestern Atlantic reef fish assemblages, where it is predominantly associated with deeper reef habitats and mesophotic environments (Silva et al., 2021).

**TABLE 1:** Summary of fish surveys, fisheries monitoring and data compilations conducted in Baía de Todos os Santos (BTS) and the adjacent continental shelf between 1973 and 2024, highlighting the absence of *Rhomboplites aurorubens* and *Centropyge aurantonotus* across all sources.

Type of study	Temporal coverage	Spatial coverage	Scientific collections consulted	Number of recorded species	Source	Remarks
Data compilation	1973–2021	Entire BTS and adjacent continental shelf	Yes	414	Oliveira‐Silva et al. (2024)	Eleven scientific collections were consulted
Data compilation	1973–2022	Entire BTS and adjacent continental shelf	No	289	Oliveira‐Silva et al. (2025)	119 peer‐reviewed articles and two books were consulted
Aquarium fishery landings	1996–2005	Entire BTS	No	197	Reis‐Filho et al. (2018)	6968 landings examined
Research captures and underwater censuses	2014–2024	Aratu bay (inner to BTS)	No	109	Medeiros et al. (2024)	Biannual campaigns
Underwater visual censuses (via BRUVs)	2014–2024	Entire BTS and adjacent continental shelf	No	191	Reis‐Filho (in prep)	914 BRUV deployments
Underwater visual censuses (via BRUVs)	2014	Entire BTS and adjacent continental shelf	No	119	Reis‐Filho et al. (2019)	Intensive sampling on the continental shelf
Fishery landings	2012–2015	Western sector of the BTS	No	178	Reis‐Filho et al. (2018)	77,863 fishery landings examined
Underwater visual censuses	2007–2008	Mouth of the BTS	No	76	Ferreira et al. (2015)	
Research captures	2004–2005	Northern sector of BTS	No	44	Dias et al. (2011)	

Abbreviations: BTS, Baía de Todos os Santos; BRUV, Baited remote underwater video.

To further substantiate these findings and to advance a mechanistic understanding of the process underlying these species' unusual occurrence in shallow waters, we synthesize multiple independent lines of evidence. These include fisheries‐based records from the adjacent continental shelf, which attest to the recurrent presence, particularly of the vermilion snapper in nearby mesophotic environments; spatially and temporally resolved temperature data spanning both the shelf and the interior of the bay, capturing periods of upwelling‐driven thermal intrusion; and a comprehensive compilation of primary and secondary fish inventories conducted over multiple decades, which collectively corroborate the novelty of these records within BTS. By integrating these complementary data streams, we move beyond simple occurrence reporting to evaluate whether transient thermal connectivity can facilitate cross‐shelf incursions of mesophotic‐associated fishes into shallow tropical coral reef systems. In doing so, this study contributes to a broader understanding of mesophotic‐euphotic coupling, ecological permeability across shelf gradients and the dynamic processes that intermittently relax otherwise persistent environmental filters structuring tropical reef fish assemblages.

First, leveraging a network of small‐scale and recreational fishers integrated into the long‐term ecological monitoring programme operating in BTS (i.e., the Long‐Term Ecological Research Program of the Baía de Todos os Santos—PELD‐BTS), we compiled information on the occurrence, fishing grounds and sporadic catch volumes of the vermilion snapper along the adjacent continental shelf directly offshore of the bay. These fishers operate across a broad range of coastal habitats, including shallow areas and deeper shelf environments; however, within this broader fishing domain, records of the vermilion snapper were consistently associated only with deeper offshore fishing grounds. This collaborative fisher‐researcher network has previously proven effective for addressing scientific questions in the region, including the identification of spawning aggregation sites of permit and pompano species (see Reis‐Filho et al., 2021), and provides a reliable framework for tracking spatially explicit fisheries information. Through this approach, we identified the principal fishing grounds where the vermilion snapper is recurrently captured (Figure 1), all located outside BTS at depths exceeding 40 m and corresponding to mesophotic habitats. When contrasted with our review of the ichthyofauna documented for BTS (Table 1), this reinforces the conclusion that the species has been historically associated with shelf environments rather than shallow habitats inside the bay.

**FIGURE 1:**
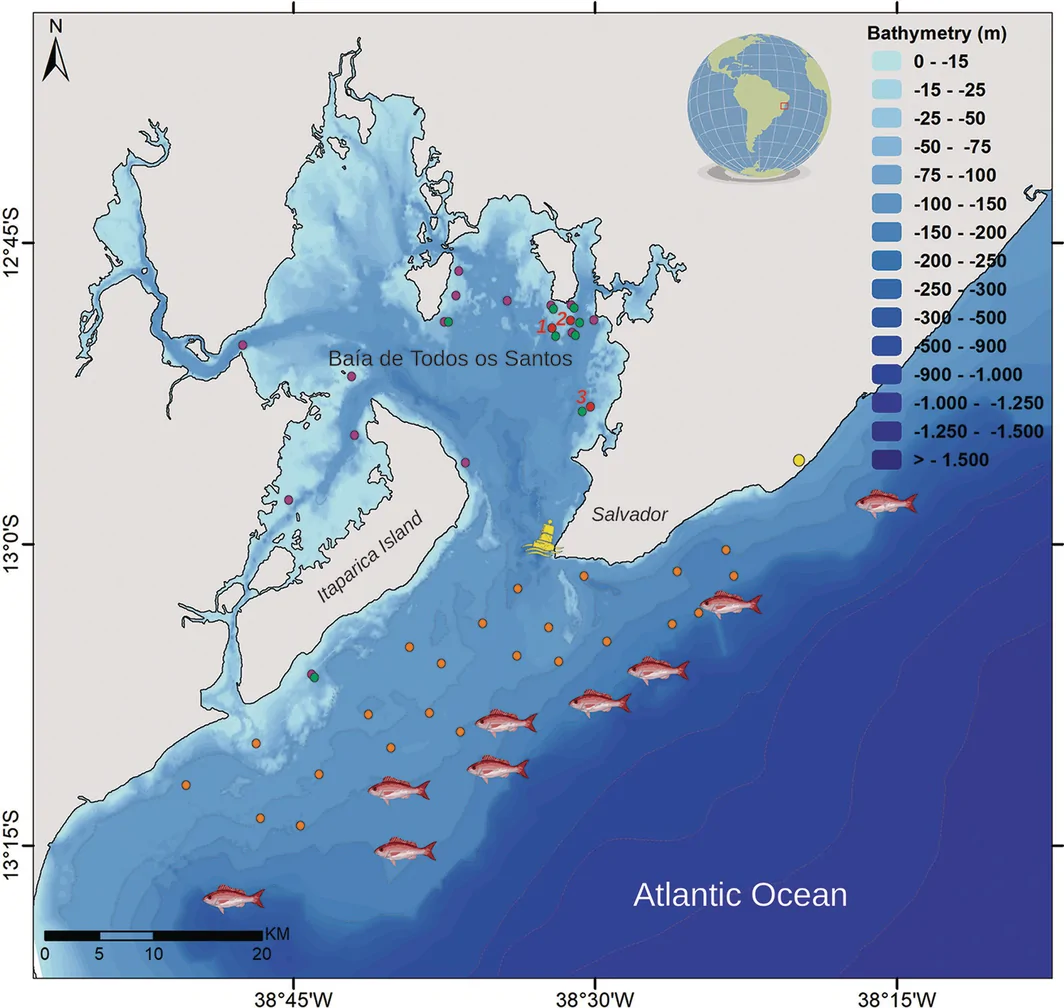
Location of Baía de Todos os Santos (BTS) and bathymetry of the adjacent continental shelf. Red circles indicate the shallow coral reef sites where *Rhomboplites aurorubens* (2,3) and *Centropyge aurantonotus* (1) were recorded. Named as: (1) Pedra Cardinal; (2) Pedra do Dentão; and (3) Pedras Alvas reefs. Purples circles indicate Baited remote underwater video system (BRUVs) sampling sites and green circles indicate underwater visual census sites from the PELD‐BTS long‐term ecological research programme. Orange circles indicate BRUV deployments on the adjacent continental shelf extracted from Reis‐Filho et al. (2019). Yellow symbols indicate the location of the SiMCosta buoy at the BTS entrance channel, the bottom mooring at the shelf and the meteorological station at the Salvador Airport. Red fish symbols indicate mesophotic fishing grounds (>50 m) where *R. aurorubens* is recurrently captured by artisanal and recreational fisheries outside the bay.

Fishery records help to identify the main fishing grounds where the vermilion snapper is recurrently captured (Figure 1) and indicate that this species has been consistently targeted outside BTS over time (Figure 2 and Figure 3a). This species has long been a common component of snapper‐directed fisheries, in which both artisanal and recreational sectors have historically captured it only in deeper offshore fishing grounds, at depths exceeding 40 m, corresponding to mesophotic habitats. Because fisher‐derived catch data are not standardized by effort or sampling frequency, these records are used here to document recurrent occurrence and capture outside BTS rather than to estimate catch rates or formally evaluate temporal changes in abundance.

**FIGURE 2:**
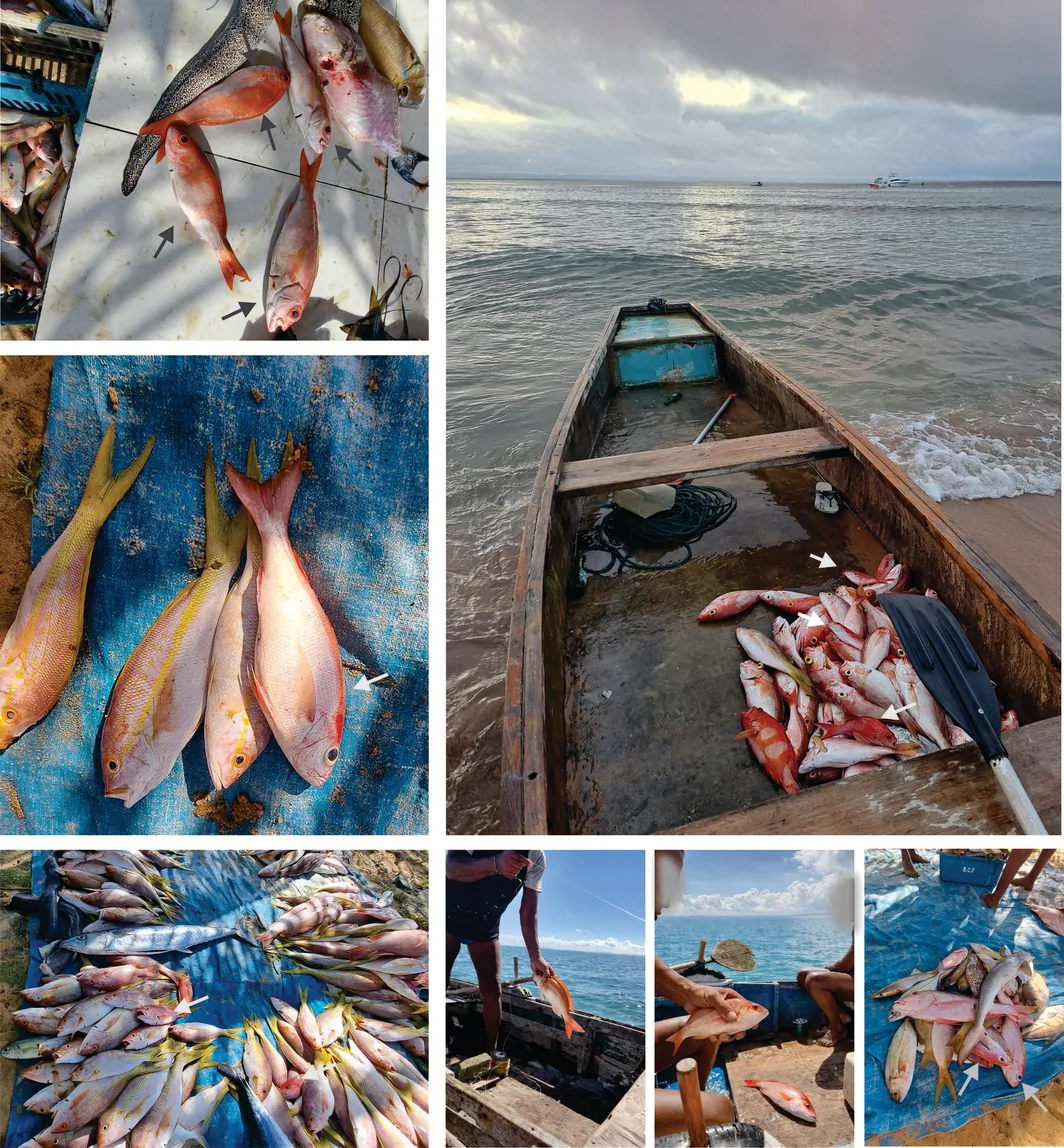
Photographic records documenting the recurrent capture of the vermilion snapper *Rhomboplites aurorubens* by artisanal and recreational fisheries along the continental shelf adjacent to Baía de Todos os Santos. The images show captures consistently obtained in offshore waters and mesophotic fishing grounds (>40 m depth), frequently in association with other snapper species, reinforcing the regular occurrence of this species outside the bay. Arrows indicate individuals of *R. aurorubens* within multi‐species catches.

**FIGURE 3:**
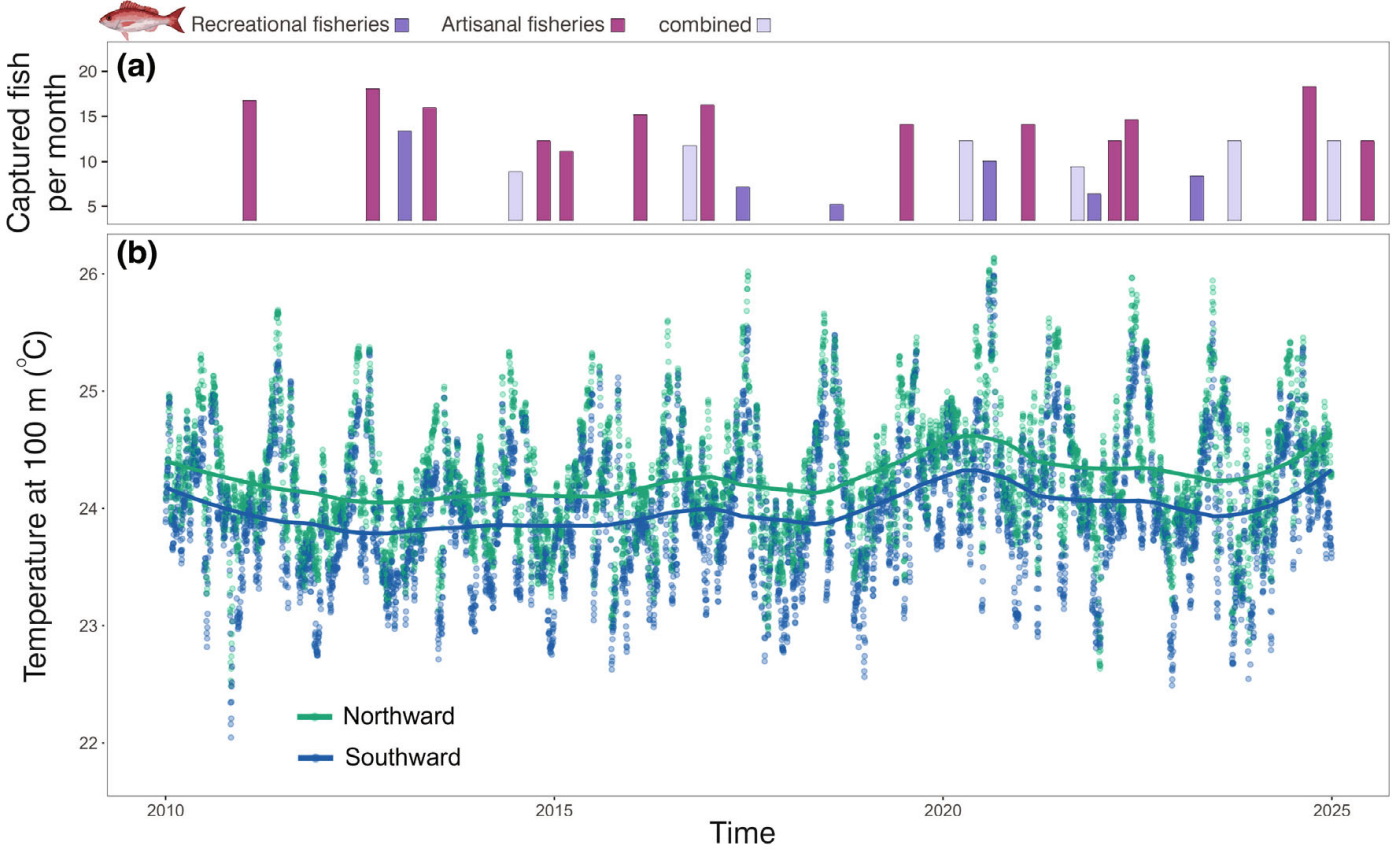
Descriptive temporal context of vermilion snapper (*Rhomboplites aurorubens*) records along the continental shelf adjacent to Baía de Todos os Santos and associated subsurface thermal conditions. (a) Monthly number of individuals reported in fisher‐derived records from artisanal fisheries, recreational fisheries or combined, based on the collaborative monitoring network. These records are not standardized by fishing effort and are used only to document the recurrent capture of the species outside BTS over time; months without bars should not be interpreted as confirmed absences. (b) Time series of seawater temperature at 100 m depth extracted from Copernicus Marine Service reanalysis products for the main offshore fishing grounds located northward and southward of the bay entrance. Solid lines represent smoothed temporal trends for each sector, illustrating that these deeper shelf habitats are consistently associated with relatively low subsurface temperatures compared with typical shallow bay conditions, while also capturing episodic cooling events.

The thermal regime of these offshore fishing grounds was characterized using seawater temperature at 100 m deep (Figure 3b). The data were obtained from the Global Ocean Physics Reanalysis product from the Copernicus Marine Environment Monitoring Service (CMEMS) between 2010 and 2024, a period with available fishery records (https://marine.copernicus.eu). This reanalysis product provides gridded oceanographic estimates derived from numerical ocean models constrained by observations, allowing the characterization of subsurface thermal variability at the fishing‐ground scale. The mean temperature at that depth was 24°C, but it exhibited consistent seasonal oscillations, with average minimum temperatures during spring–summer being 1.05°C lower than the average winter maximum temperatures. These colder spring–summer temperatures are associated with wind‐forced upwelling, a meso‐scale process that brings colder subsurface waters upward along the continental slope over at least an 80‐km‐long coastal segment northward of the BTS entrance (Thévenin et al., 2019). Although a formal correlation between catches and temperature along the shelf margin cannot be established, these data still provide a reliable characterization of the thermal envelope associated with mesophotic fishing grounds where the vermilion snapper is commonly captured along the continental shelf adjacent to BTS.

Sampling using baited remote underwater video systems (BRUVs), with approximately 1 h 30 min of soak time per deployment, has been conducted consistently in BTS since 2014 at six‐month intervals, with three replicates per monitoring site. BRUVS deployments were conducted using small action cameras mounted inside underwater housings, specifically GoPro HERO12 Black® cameras. Diver‐based underwater visual censuses following the same temporal schedule have been conducted from 2022 to the present. BRUVS deployments and diver‐based visual censuses were not conducted simultaneously or on the same day, but within the same sampling week, avoiding potential interference between methods. In November 2024, a single individual of the pygmy angelfish *C. aurantonotus* was recorded at Pedra Cardinal Reef (reef site 1 in Figure 1) during human‐based underwater visual census (Figure 4g). Subsequently, *R. aurorubens* was observed in June 2025 at Pedras Alvas Reef (reef site 3), where a small group of four juvenile individuals (~15 cm total length) was recorded during visual census (i.e., human‐based) (Figure 4f,h). Additional BRUV records of *R. aurorubens* were obtained in April and December 2025 at Pedra Cardinal, Pedras Alvas and Pedra do Dentão (reef site 2) reefs, where individuals occurred in small aggregations of three to five fishes between 15 and 20 cm in total length (Figure 4a–e). Taken together, these records indicate that both species likely travelled about 40 km inshore from their mesophotic realm at the shelf's edge. *C. aurantonotus* was readily distinguished by its deep‐bodied profile, intense cobalt‐blue coloration over the anterior body and bright orange posterior region extending from the mid‐body to the caudal peduncle, a unique colour pattern among southwestern Atlantic angelfishes (Floeter et al., 2023). Juvenile *R. aurorubens* were identified by their elongate lutjanid body shape, uniformly reddish coloration with subtle silvery hues, large eyes and the absence of horizontal striping or ocelli typical of other sympatric juvenile snappers, consistent with descriptions for early life stages of the species (Floeter et al., 2023).

**FIGURE 4:**
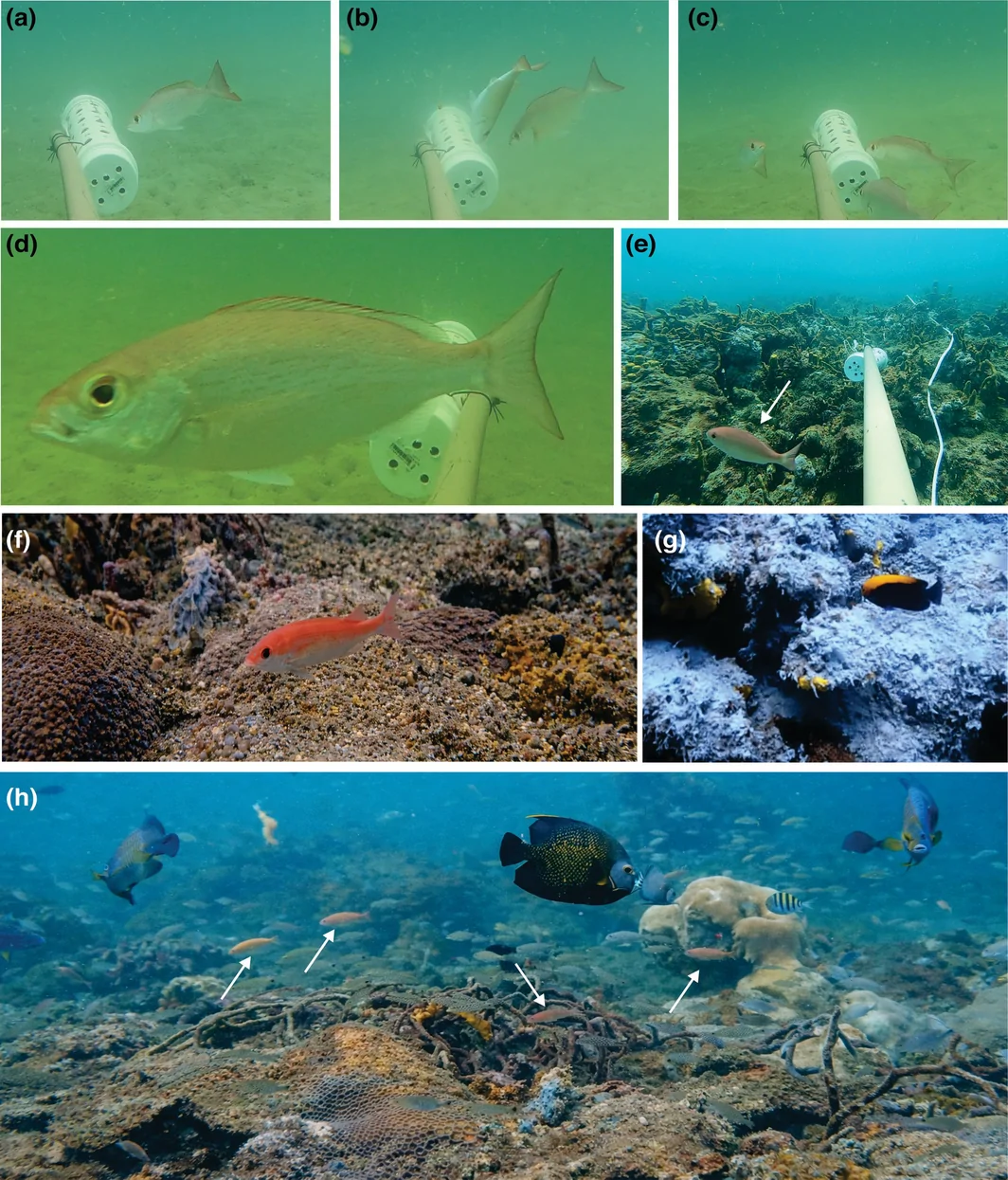
Images documenting the first occurrences of mesophotic‐associated fishes inside Baía de Todos os Santos (BTS). (a–e) Baited remote underwater video (BRUV) snapshots of juvenile *Rhomboplites aurorubens* (Pedras Alvas Reef). (f and h) Diver‐based photographs of *R. aurorubens* (arrow) (Pedras Alvas and Pedra do Dentão reefs, respectively). (g) Photograph of *Centropyge aurantonotus* (Pedra Cardinal Reef). Credits: a–e, José Amorim Reis‐Filho; f–h, Rodrigo Maia Nogueira.

Although the records of these deep‐associated species inside BTS are temporally limited, they represent the first documented occurrences of *R. aurorubens* and *C. aurantonotus* within this exceptionally well‐studied tropical bay, where long‐term monitoring datasets exist (Table 1). Notably, with the exception of June 2025, the unprecedented records occurred within a window of influence of an upwelling event. Figure 5 shows a time series of the surface temperature at the BTS entrance channel (see Figure 1) recorded by the SiMCosta buoy BA‐1 (http://simcosta.furg.br), the bottom temperature on the shelf at 32 m deep recorded by the SiMCosta mooring BA‐2, the alongshore wind component recorded at Salvador International Airport, station SBSB at the ASOS‐AWOS‐METAR network (https://mesonet.agron.iastate.edu) and the days of fish sightings (red arrows). Surface‐to‐bottom temperature differences occur in the spring and summer when the alongshore wind component is negative, indicating southbound alongshore winds, forcing an offshore surface Ekman transport and a seasonal upwelling (Thévenin et al., 2019). Stronger upwelling events, associated with accelerating negative alongshore wind components, are punctuated by temperature depressions that last 3–5 days on the shelf and are replicated at the surface at the inlet channel. The November 2024 and December 2025 fish records are associated with negative alongshore wind components, indicating southward alongshore winds, and occurred shortly after the coolest temperatures were registered, whereas the April 2025 record took place during a cooling event, prior to the coolest temperature on the shelf. The conveyance of colder shelf waters into the bay is facilitated by the estuarine gravitational circulation that is established along the deep (>20 m deep) channels, a process well documented by Lessa et al. (2019) and Fonseca et al. (2024). It provides a plausible mechanistic context for the observed occurrences of *R. aurorubens* and *C. aurantonotus* 20 km into the BTS and at least 40 km inshore of mesophotic sites.

**FIGURE 5:**
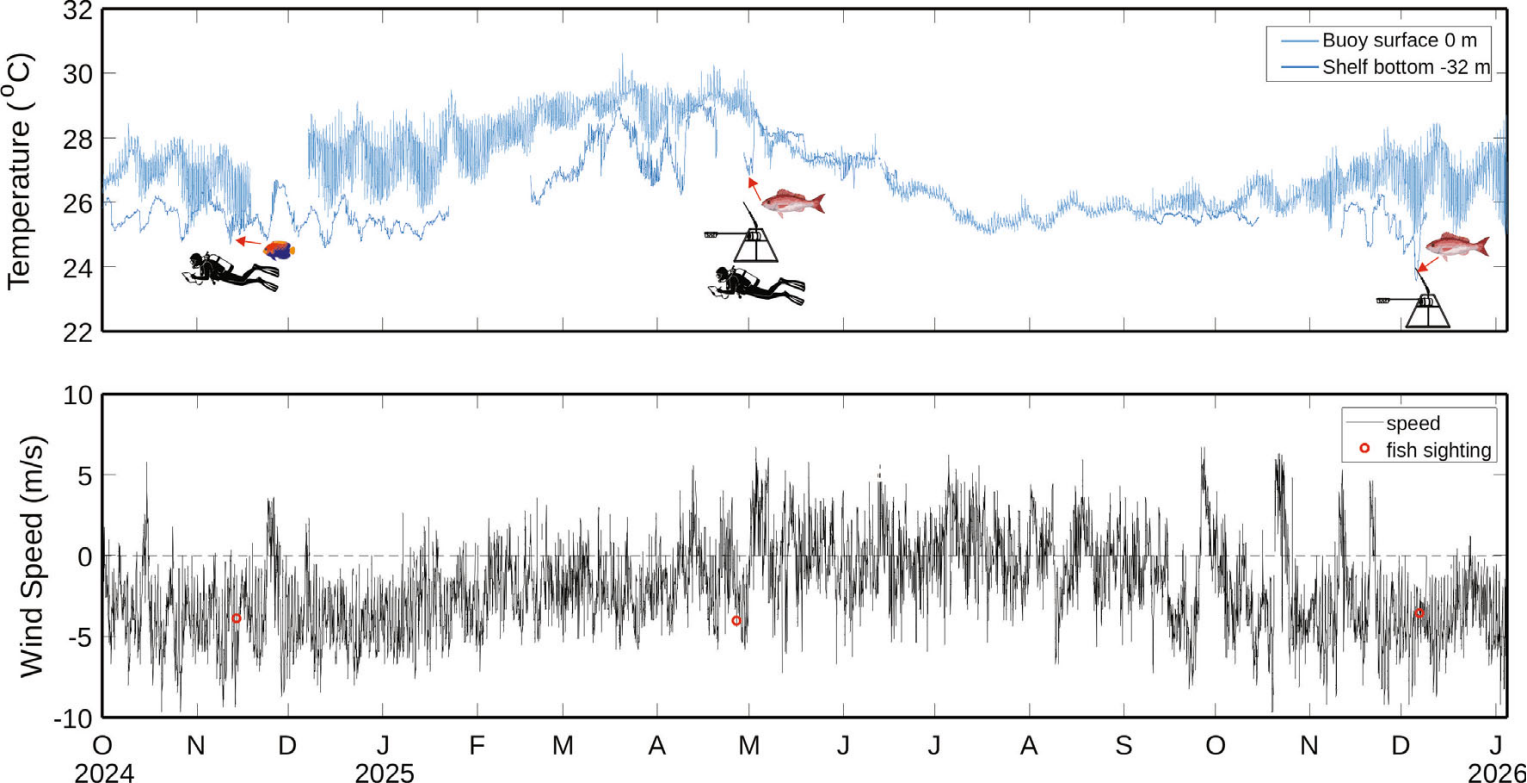
High‐resolution time series of seawater temperature and the alongshore wind component. Water temperatures were recorded by SiMCosta moorings at the surface at the entrance of Baía de Todos os Santos and at a 32‐m‐deep shelf setting. Alongshore wind speed (i.e., negative values indicate southerly direction) is shown to illustrate seasonal thermal variability and episodic cooling events associated with upwelling dynamics. Icons in the upper panel indicate the timing of ecological monitoring programme (Baited remote underwater video [BRUV] deployments). Dashed red circles highlight periods during which *Centropyge aurantonotus* and juvenile *Rhomboplites aurorubens* were detected inside the bay, coinciding with phases of reduced water temperature.

The detection of these species under well‐defined thermal conditions supports the hypothesis that transient cold‐water incursions may function as ecological corridors, temporarily relaxing thermal barriers and enabling mesophotic‐associated fishes to access shallow reef habitats that are otherwise thermally unsuitable (Silva et al., 2025). Furthermore, light attenuation resulting from frequent sediment suspension—typical of embayments such as BTS (Devlin et al., 2008)—may synergistically interact with cooler water masses to create conditions that facilitate the occupation of shallow habitats by mesophotic‐associated fishes, as reported for the subtropical southwestern Atlantic (Gragnolati et al., 2024). In this sense, these records are unlikely to represent isolated biogeographical anomalies but instead suggest a dynamic and climate‐sensitive coupling between external mesophotic environments and inner‐bay reef systems. We acknowledge that such cross‐shelf movements may occur over short temporal windows and potentially at higher frequencies than those captured by our sampling design, even within a well‐resolved monitoring framework. Nevertheless, the observed synchronicity between fish sightings and cooling events cannot be disregarded. It is also plausible that individuals may return to deeper, colder mesophotic habitats once cold‐water incursions in shallow reefs subside, making these shallow habitats thermally less suitable again. However, our temporally structured but snapshot‐based sampling design does not allow us to confirm the direction, duration or reversibility of these movements.

Life‐history stage may also be relevant to interpreting these occurrences. The *R. aurorubens* individuals recorded inside BTS were juveniles or small individuals, whereas the single *C. aurantonotus* record does not allow inference on population establishment. Ontogenetic habitat shifts and shallower juvenile habitats are known for some snappers, including *Pristipomoides filamentosus* (Moffitt & Parrish, 1996). Similarly, tropical vagrants transported by boundary currents, such as those carried poleward by the East Australian Current, may persist temporarily without necessarily establishing reproductive populations (Booth et al., 2007; Figueira & Booth, 2010; Vergés et al., 2016). Thus, these records are best interpreted as transient occurrences rather than evidence of local population establishment.

Importantly, the detection of these events was only possible because of a well‐structured, long‐term and temporally persistent ecological monitoring programme. This underscores the critical value of sustained observational frameworks with dedicated funding, without which rare, episodic, yet ecologically meaningful processes would likely remain undetected (Cordeiro et al., 2022). Finally, although still preliminary, these findings raise compelling questions in the context of ongoing ocean warming (Munday et al., 2008, 2009). Seasonal upwelling‐driven cooling events may locally attenuate warming trends that disproportionately affect shallow reef systems (Izquierdo et al., 2022), while simultaneously facilitating cross‐shelf connectivity and habitat use by mesophotic‐associated species. However, the expected increase in the frequency and duration of upwelling regimes under climate change scenarios can modify local nutrient dynamics and directly affect productivity and habitat suitability for multiple species (Bakun et al., 2015; Wang et al., 2015). Understanding the frequency, duration and ecological consequences of such processes will be important under future climate scenarios.

## AUTHOR CONTRIBUTIONS

**José Amorim Reis‐Filho:** conceptualization, data collection, formal analysis, writing—original draft, writing—review, edition. **Miguel Loiola:** data collection, writing—review, edition. **Rodrigo Maia‐Nogueira:** data collection, writing—review. **Carla Sthefany Barroso Teles:** writing—review. **Maisha Gragnolati:** writing—review. **Guilherme Camargo Lessa:** writing—review, formal analysis, writing—review. **Pedro Milet Meirelles:** writing—review. **Igor Cristino Silva Cruz:** writing—review.

## FUNDING INFORMATION

Financial support was provided by Long‐Term Ecological Research Program PELD‐BTS (CNPq 445990/2024–4).

## CONFLICT OF INTEREST STATEMENT

The authors declare no conflicts of interest.

## Data Availability

The data that support the findings of this study are available from the corresponding author upon reasonable request.
